# Digital transformation in restaurants: key aspects of service robot deployment from project initiation to evaluation

**DOI:** 10.3389/frobt.2026.1793138

**Published:** 2026-04-22

**Authors:** Anniken Susanne T. Karlsen, Bjørn Andersen, Solvår Elverum Heirsaunet, Elin Indergård, Kristina Nevstad, Wenche Aarseth

**Affiliations:** 1 NTNU Norwegian University of Science and Technology, Trondheim, Norway; 2 NORD University Business School, Bodø, Norway

**Keywords:** digital transformation, project evaluation, project execution, project initiation, project planning, restaurants, service robots, technology deployment

## Abstract

This study examines the deployment of service robots designed to support waitstaff in food delivery within Norwegian restaurants, which are national pioneers in adopting this technology. It investigates key aspects of service robot deployment, from project initiation through evaluation, and the approach used to ensure that robot functionality aligns with restaurant workflows and spatial configurations. Using an exploratory–explanatory case study design, the research draws on 22 interviews with 34 participants, complemented by observational fieldwork to strengthen contextual understanding. The findings offer an integrated view of the digital transformation process, identifying important considerations across all project phases. One example is the importance of considering incorporating robot service needs into early facility planning. By addressing potential obstacles such as stairs and doorsteps during the front-end design, restaurants can avoid costly redesigns and ensure optimal robot performance. By examining real-world deployment, the study offers practical insights for project managers, hospitality leaders, and others preparing to integrate service robots into restaurant operations.

## 1 Introduction

According to Intel Market Research (2026), the global hospitality service-robot market was valued at USD 1.02 billion in 2025 and is projected to reach USD 3.45 billion by 2034. These figures indicate a deeper digital transformation in the operational practices of hotels and restaurants, where growth is driven by persistent labor shortages, estimated at more than 1.5 million unfilled positions worldwide, along with rising demand for contactless services and advances in robotics technology (Intel Market Research, 2026). In this context, service robots are increasingly adopted to boost productivity and maintain service levels (Collins, 2020; Tuomi et al., 2021b; Choi and Wan, 2021; Mukherjee et al., 2023; Ivanov and Webster, 2019a; Tan et al., 2025; Intel Market Research, 2026), particularly for labor-intensive or repetitive tasks (Lu et al., 2020; Xu et al., 2023; Ivanov and Webster, 2019a; Banu et al., 2025; Intel Market Research, 2026). Intel Market Research (2026) also identifies several barriers to broader adoption. High upfront investment remains a central obstacle, with advanced units priced at USD 20,000–100,000. Additional challenges stem from the need to integrate robots with existing, often outdated, hospitality systems, as well as from maintenance requirements that can lead to three to 5 days of downtime. Together, these factors limit the feasibility of robotics implementation for many operators (Intel Market Research, 2026).

Although the development and application of service-robot technologies have advanced substantially in hospitality, and the market continues to expand at a rapid pace, important research gaps remain. First, much of the existing literature concentrates on technology acceptance or customer perceptions, providing only limited insight into what actually occurs during real-world deployments of service robots in operational settings. As noted by Xu et al. (2023) and Madhan et al. (2023), limited attention has been given to extracting practical insights from real-world deployments of service robots (Xu et al., 2023; Madhan et al., 2023). Second, there is a lack of integrated perspectives that encompass both the pre-adoption and post-adoption phases (Cho and Kwon, 2025), i.e., that span the complete set of project phases. This absence of a holistic project perspective echoes the broader gap identified by Takagi et al. (2025) in the existing literature on the management of digital transformation projects, as well as Ngereja et al.’s (2024) observation that empirical, process-oriented analyses of digitalization initiatives are still underdeveloped, calling for more in-depth and comprehensive analyses. Third, the existing body of research is geographically concentrated, limiting diversity in contextual understanding. A structured Web of Science and Scopus search (June and December 2025) using terms related to robots, restaurants, and case studies showed that the majority of published research originates from the People’s Republic of China and the United States. While this aspect is not the primary basis for identifying the research gap, it supports the observation that empirical accounts from other regions are underrepresented. As a result, current knowledge does not adequately capture how for instance cultural, regulatory, organizational, or labor-market differences may influence service robot deployment.

Taken together, these points reveal a distinct research gap: the field lacks holistic and contextually varied case studies that follow service robot deployment across all major project phases, particularly from geographical regions that remain underrepresented in the existing literature. Motivated by this gap, we present findings from a Norwegian case study that examines the deployment of delivery robots to support waitstaff. The study covers all major project phases, including initiation, planning, execution, and evaluation, and is conducted in restaurants that are national pioneers in the adoption of this technology. In doing so, we aim to strengthen the existing body of knowledge by providing insights into key aspects of service robot deployment from a different contextual setting. In addition, ongoing staffing challenges in Norway’s restaurant industry (Myklathun and Skjøstad, 2024) further underscore the relevance and practical significance of this investigation. The use of a case study approach is supported by Yin (2011), who argues that case studies can generate deeper insights by preserving the holistic and meaningful characteristics of real-world phenomena.

Our research was guided by the following questions.

RQ1 What are the key aspects of service robot deployment, from project initiation through evaluation?
RQ2 What approach is used to ensure that robot functionality aligns with restaurant workflows and spatial configurations?

Section 2 establishes a foundation for the topic under investigation. Section 3 details our research methodology. Section 4 presents our findings. In Section 5, we discuss these findings, and in Section 6, we draw conclusions and offer suggestions for future research.

## 2 Theoretical background: technology deployment

### 2.1 What is a service robot?

According to the International Organization for Standardization (ISO, 2021), a robot is a programmed and actuated mechanism that possesses a degree of autonomy enabling it to perform locomotion, manipulation, or positioning tasks. The same standard defines a service robot as a robot used either for personal or professional purposes to carry out useful tasks for humans or equipment. This broad definition distinguishes service robots from industrial robots, which are designed primarily for automated manufacturing contexts. Aligning with this distinction Habib (2006) define a service robot as a generic term covering all robots that are not intended for industrial use. Wirtz et al. (2018) offer a more specific conceptualization tailored to frontline service environments. They define service robots as system-based, autonomous, and adaptable interfaces that interact and communicate with customers while delivering services on behalf of an organization. This perspective highlights the role of service robots not only as task performers but also as participants in the service encounter.

### 2.2 Use of service robots in hospitality

Historically, robots have primarily been used for industrial purposes (Nedelkoska and Quintini, 2018; Pocriciuc, 2023; Urrea and Kern, 2025). In recent years, however, their use has expanded significantly, with robots increasingly deployed across enterprises and private households to provide a wide range of services (Tuomi et al., 2021b; Gonzalez-Aguirre et al., 2021; Paluch et al., 2020; Chiang and Trimi, 2020; Jia et al., 2024; Yoganathan et al., 2025). This broader adoption is driven by an increasing variety of service robot types developed and made available (De Keyser and Kunz, 2022; Yoganathan et al., 2025; Ivanov and Webster, 2019a), such as vacuum cleaning robots (Prassler et al., 2000; Tanaka et al., 2025), hospital assistants (Ahn et al., 2015; Kyrarini et al., 2021; Radic et al., 2022; Ivanov and Webster, 2019a; Gasteiger et al., 2025), personal care robots (Bilyea et al., 2017; Fetterolf et al., 2025), and coaching robots (Sackl et al., 2022; Axelsson et al., 2025).

Within the hospitality sector, service robots now support a broad range of operational tasks, including front-desk assistance, cleaning, guest-room deliveries, information provision, and other automated processes that enhance efficiency and productivity (Banu et al., 2025). Their deployment spans both hotels and food-and-beverage settings. Hotels, for instance, use robots for room service, programming them to navigate hallways and elevators, knock on guest doors, and deliver meals, beverages, or amenities (Banu et al., 2025). When combined with other technologies, robots also enable more advanced and integrated service delivery (Ivanov and Webster, 2019a; Talukder, 2025). These technological developments are highly relevant to restaurants, which since the COVID-19 pandemic have struggled with staffing shortages, customer retention, rising inflation-related costs, and widespread closures and bankruptcies (Olsson, 2024). To address such challenges, restaurants for instance make use of service robots to take orders (Seyitoğlu et al., 2021; Banu et al., 2025), to serve food and drinks, and to clear tables (Banu et al., 2025). Evidently, service robots have evolved beyond their initial novelty status and now fulfill substantive, practical functions in the hospitality industry (Banu et al., 2025).

### 2.3 Technology deployment aspects across project stages

Effective project management is vital for the successful deployment of technology (Baškarada et al., 2013). Projects can be structured in different ways, and the number of phases typically depends on the chosen project management methodology, with four- and five-phase models being common. In the context of technology deployment, Baškarada et al. (2013) groups the deployment activities roughly into four stages: initiation, planning, execution, and evaluation. In what follows, we examine key aspects of technology deployment identified in the literature, organized according to these stages to align with our focus on service robot deployment.

***The initiation stage*** begins when prospective users develop a business case that outlines a capability gap, improvement opportunity, operational urgency, and other relevant aspects (Baškarada et al., 2013). Despite the importance of front-end management in projects, there are several paradoxes. For example, fewer resources are allocated upfront to identify the best conceptual solution, such as designing a restaurant project to efficiently utilize service robots, compared to the resources used to improve tactical performance during implementation (Samset and Volden, 2016).

From the earliest stages of a project, it is important to identify and understand potential sources of resistance. Doing so enables project managers to address these challenges effectively by isolating, mitigating, and even transforming resistance into constructive forces (Berna-Martinez and Maciá Pérez, 2012). In the context of service robots, it is essential to develop a thorough understanding of the factors that influence people’s acceptance of this technology, as well as the underlying reasons for both adoption and resistance. Over the years, a range of models and theories have been developed to explore these aspects of technology adoption, including those proposed by Davis (1989), Moore (1989), Thompson et al. (1991), Igbaria et al. (1996), Wang et al. (2010), Venkatesh et al. (2016), and Ibrahim et al. (2025). Among these models, the Technology Acceptance Model (TAM) by Davis (1989) is widely recognized as one of the most influential. In the book “The technology acceptance model: 30 years of TAM”, Davis et al. (2024) conclude that TAM is a simple, powerful, and well-validated, theory-based model. It centers on perceived usefulness and perceived ease of use, both influenced by system design features. These perceptions shape user attitudes and intentions, which ultimately determine technology acceptance. According to the authors, TAM therefore predicts acceptance, explains why users adopt or reject a system, and offers guidance for improving acceptance through design or managerial interventions. The authors emphasize that although many extensions have been added over the past 3 decades, perceived usefulness and perceived ease of use remain the core of the model (Davis et al., 2024).

In addition to various models and theories on technology adoption, many articles exist, reporting on the advantages and disadvantages, conditions, challenges, and attitudes both for and against adopting service robots in hospitality. For instance, in a study investigating the perception of restaurant robots among general managers or deputy managers in more than hundred restaurants in Taiwan, findings indicated among others that perceived usefulness and perceived ease of use influences the attitude towards the technology (Lee et al., 2018). Based on their findings of issues related to the use of robots, artificial intelligence, and service automation in travel, tourism, and hospitality, Ivanov and Webster (2019a) developed a conceptual framework. At the core of the framework is a travel, tourism, and hospitality company, which has two layers of stakeholders, i.e., internal (employers and managers) and external (robots, artificial intelligence, and service automation suppliers, competitors, and customers). In their work several key aspects of adoption, including driving factors, the comparative advantages, and disadvantages of robots, artificial intelligence, and service automation technologies *versus* human employees, managerial decision-making processes, and the impacts on business operations, are explored. A notable emphasis is placed on how macroenvironmental pressures influence microeconomic decisions to implement robots, artificial intelligence, and service automation within the travel, tourism, and hospitality context (Ivanov and Webster, 2019a). A study by Yoganathan et al. (2025) also address the importance of understanding attitudes towards service robots at the population level. Among others they find, as service robots become more mainstream, that from a managerial perspective there is greater need for firms to acknowledge and cater to different population-level attitudes. Ho and Chuah (2025) surveyed one hundred and two staff members in Malaysian restaurants expected to introduce service robots. They examined how drivers (e.g., management support, relative advantage, enjoyment), barriers (e.g., technical issues, job insecurity, privacy risks), and employee characteristics (e.g., social skills, openness to experience) interact to explain employees’ intention to work with service robots. Among others, their findings suggest that employees open to new experiences and with strong social skills are likely to work with service robots if they perceive high relative advantage and enjoyment and feel low job insecurity. Additionally, employees with strong social skills show high intention to work with service robots when supported by management, despite technical concerns, job insecurity, and privacy issues (Ho and Chuah, 2025).

According to Lee (2018) the adoption of robots in business operations is typically shaped by external factors, including customer expectations and demands, as well as demographic changes such as declining birthrates and an increasing elderly population. The latter calls for technology advancements to alleviate the resulting labor shortages (Lee, 2018; Omori, 2008; Pedersen et al., 2018). A factor attributed to heightened interest in service robots is the impact of the COVID-19 pandemic making consumers aware of the risk of infectious diseases from interpersonal interactions (Choi and Wan, 2021; Wan et al., 2021; Kang et al., 2022). In fact, in certain situations the pandemic shall have evoked perceptions making customers prefer service robots over human staff (Kim et al., 2021), going from being viewed as a threat to being seen as contributors to managing future crises, as evidenced by research by Ghafurian et al. (2021), as well as Shen et al. (2020), and insights from OECD (2021). Generally, while some people are willing to adopt a new technology rapidly, others resist, depending on the perceived characteristics of the innovation (Choi et al., 2020). Guests are generally receptive to the use of service robots, regardless of their country of origin (Ivanov et al., 2018; Seyitoğlu et al., 2021). While Seyitoğlu et al. (2021) report that employees and managers in restaurants tend to have a predominantly negative attitude towards the use of robots, Vatan and Dogan (2021) find hotel employees believing service robots may provide various benefits and advantages for employees and businesses. A generational difference in attitudes towards robots also exists, whereby Generation X (born 1960–1980) shows less interest in experiencing robots compared to Generation Y (born 1980–2000), and Generation Z (born 1990–2010) (Ayyildiz et al., 2022) – regarded as the Internet generation (Tapscott, 1998; Borg et al., 2023). Generally, the attitude towards robots is influenced by variables such as class, wealth, and education (Ivanov et al., 2018). Interestingly, people’s preference, acceptance, and interaction with robots may increase when they perceive them to exhibit more human-like qualities (Stroessner and Benitez, 2019). One example of a humanoid robot, i.e., a robot designed with anthropomorphic qualities, meaning it has been given human-like physical or non-physical characteristics (Salles et al., 2020; Guthrie, 1997; Pfeuffer et al., 2019), is Softbank Robotics’ robot named “Pepper”. This is a popular service robot managing visitor encounters in hotels, restaurants, and retail establishments (Mingotto et al., 2021; Tuomi et al., 2021a). In certain situations, customers may feel less embarrassed when interacting with a robot like “Pepper” since it is devoid of emotions and refrains from making moral or social assessments (Pitardi et al., 2021).

***The planning stage*** centers on assessing how new technologies may influence existing procedures and operational routines (Baškarada et al., 2013). Brown (2022) argues that the successful engineering of systems incorporating intelligent technologies requires integrated, cross-disciplinary perspectives, along with tools that support a shared understanding among stakeholders regarding intended outcomes. From an economic standpoint, planning for the adoption of assets such as service robots must also be financially justified. Such investments are warranted only when they are expected to enhance a company’s financial performance and create value for shareholders (McLaney et al., 2007; Berk and DeMarzo, 2017; Ivanov and Webster, 2019b). Without a positive financial impact, there is little economic incentive for organizations to pursue these technologies (Ivanov and Webster, 2019b). Recent studies reinforce this rationale. Skubis et al. (2024) find that service robots are introduced to enhance operational efficiency while maintaining hospitality standards, and Belanche et al. (2021) similarly report that many firms adopt service robots to reduce operational costs and enhance customer service.

***The execution stage*** represents the phase in which a technology is implemented and put into practical use (Baškarada et al., 2013). A well-known example is the Henn Na Hotel in Japan, where service robots have been deployed for cleaning, luggage handling, voice-activated room service, and front desk operations (Granström et al., 2023; Reis et al., 2020; Bhimasta and Kuo, 2019; Sharma et al., 2023). Successfully managing technological change requires structured approaches. Techniques like agile project management have been shown to improve project outcomes by enhancing team performance and supporting future planning (Ciric Lalic et al., 2022). Similarly, enterprise modeling supports a holistic understanding of organizational processes, which is valuable in turnaround projects (Stirna and Persson, 2018; Haidar et al., 2023; Vernadat, 2020; Lantow et al., 2022; Karlsen and Opdahl, 2012). Regardless of the specific method or framework employed, clearly defining the project’s goals, budget, timeline, and the roles and responsibilities of all involved stakeholders remains essential for successful execution (Kerzner, 2017).

In addition to establishing a solid project structure, it is important to consider the functionality of the robots, the quality of human-robot interactions, and the capacity of humans and robots to collaborate effectively (Saari et al., 2022). Service robots are designed not only to automate tasks but also to enhance service quality (Fusté-Forné and Jamal, 2021). Ongoing research and development efforts have produced robots with varying levels of autonomy tailored to different hospitality functions. For example, Acosta et al. (2006) developed a prototype for setting and clearing tables, while Yano et al. (2024) introduced an autonomous waiter robot capable of recognizing customers, taking orders, and serving food. Integrating such technologies into the workplace often changes work processes and requires employees to develop new skills (Ra et al., 2019). Effective project collaboration depends on individuals who are open to learning, recognize the value of teamwork, and are willing to communicate and work toward shared objectives (Nevstad et al., 2018). As emphasized by Takagi et al. (2025), successful digital transformation relies on continuous training and the ongoing development of staff competencies.

Finally, ***the evaluation stage*** encompasses a comprehensive operational assessment, including an after-action review conducted by the project team, in order to systematically examine the effectiveness, efficiency, and overall outcomes of the project. This stage not only highlights successes and deviations from planned objectives but also facilitates the identification of lessons learned, which should be carefully documented and communicated to relevant stakeholders to support organizational learning and enhance future project performance (Baškarada et al., 2013).

## 3 Research methodology

### 3.1 Case description and choice of design

Our study aims to answer the questions: (RQ1) *What are the key aspects of service robot deployment, from project initiation through evaluation?* and (RQ2) *What approach is used to ensure that robot functionality aligns with restaurant workflows and spatial configurations?*

We adopted an exploratory–explanatory case study design to investigate a contemporary phenomenon: the deployment of delivery robots in restaurants from project initiation through evaluation. This approach is appropriate because it enables in-depth examination of a phenomenon within its real-life context (Yin, 2009; Yin, 2011), and allows us to address a *how* question that unfolds over time rather than focusing on frequency or incidence (Benbasat et al., 1987).

The study was conducted in pioneering Norwegian “fine family style” restaurants that offer a sophisticated yet warm and inclusive dining experience suitable for both special occasions and everyday family gatherings. These restaurants place strong emphasis on maintaining high-quality personal service, viewing interaction with human waiters as essential to guest experience. The service robots are therefore introduced to support, not replace, human staff, enabling waiters to devote more time to guest interaction and personalized hospitality.

Our analysis focuses on the technology-deployment project activities across the stages of initiation, planning, execution, and evaluation, as outlined by Baškarada et al. (2013).

**FIGURE 1:**
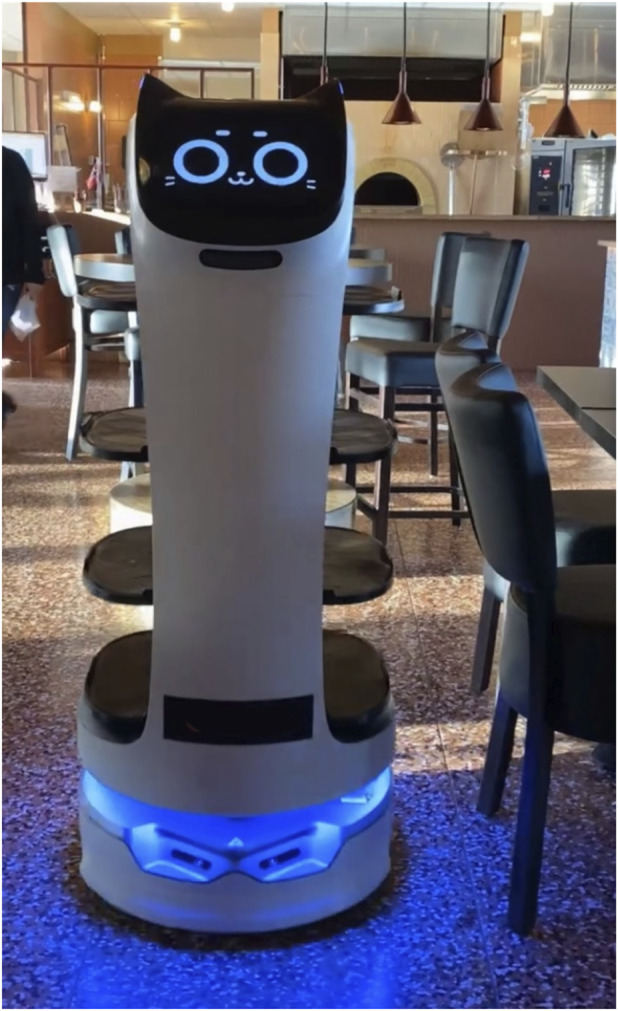
Service robot displaying a bionic facial expression; branding has been removed. Image captured by one of the authors.

The service robot examined in this study (Figure 1) is designed with anthropomorphic features, including the ability to greet guests with welcome messages and sing birthday songs. The robot moves on wheels and does not have arms. It also has a display showing a wide range of bionic facial expressions which is developed to create a warm and engaging form of human–robot interaction. It transports food using four flat induction trays with a load capacity of 40 kg and is intentionally designed to be user friendly so that waitstaff can operate it with the simple push of a button. Additional features include voice recognition and obstacle-avoidance systems.

Regarding the cost of the robot, precise pricing data has not been obtained. Nevertheless, a survey of publicly available information indicates that AI-enabled service robots designed for customer interaction and delivery functions at present typically range in price from approximately USD 20,000–30,000 to over USD 100,000, depending on the system’s technical sophistication and functional capabilities. The robot examined in our study appears to fall within the lower segment of this price spectrum. Aligned with the restaurant service philosophy, the robot is intended to function as a logistical assistant by carrying food and other items. By taking over these carrying tasks, the robot supports the primary motivation for the investment, which is to relieve staff from heavy and repetitive transport work and to enable waiters to devote more time to interacting with guests, an aspect the restaurants view as essential to delivering high-quality hospitality.

### 3.2 Data collection

Guided by a qualitative research paradigm aimed at developing a rich and comprehensive understanding of informants’ perspectives (Coleman and Briggs, 2002), we conducted interviews as conversational exchanges between interviewers and participants. Semi-structured interviews with open-ended questions were used to explore participants’ experiences with the restaurant’s new technology. This format enabled flexibility, probing, and spontaneous reflection, thereby deepening the insights generated (Easwaramoorthy and Zarinpoush, 2006). Both the interviewer and the informants adopted an open, receptive stance that encouraged free-flowing dialogue, which was essential for gaining a nuanced understanding of participants’ priorities and perspectives (Rolfe, 2006; Foley et al., 2021). The study involving human participants was reviewed and approved by Sikt, the Norwegian Agency for Shared Services in Education and Research. Participants were informed about the study’s purpose and research questions and were notified that data would be collected in accordance with qualitative ethical standards for confidentiality, anonymity, and voluntary participation, prior to providing their written consent to participate in the study. For participant recruitment, we employed a snowball sampling strategy, also known as chain-referral sampling (Parker et al., 2019), in which initially selected informants recommended additional individuals for inclusion.

Our case study consisted of two main parts. The first part focused on project initiation and planning. Here, data were collected through ten single interviews, six paired interviews, and two group interviews with four participants each. These interviews focused on how restaurant guests perceive service robots, the opportunities and challenges restaurant managers associate with their adoption, and the perspectives of project managers involved in planning and decision-making processes. In this part of the study, interview duration ranged from 5 to 130 min. Individual interviews with guests generally lasted approximately 20 min, with one notably shorter interview of about 5 min due to limited participant responses. Guest group interviews each lasted around 30 min. Individual interviews with restaurant managers and project managers typically lasted about 1 hour. One interview extended to 130 min, as the interviewee provided particularly extensive reflections on project management more broadly. Notably, the shortest interviews typically occurred with guests who had limited time during their restaurant visit, whereas longer interviews were conducted with restaurant managers and project managers who were able to provide more extensive reflections. The second part of the study examined project execution and evaluation. We conducted an in-depth interview with the robot supplier responsible for delivering and implementing the service robots. Additionally, we interviewed a restaurant owner who oversees multiple establishments using the supplier’s robots, offering broader managerial perspectives on deployment experiences. At the owner’s invitation, we also carried out an observational study at one of his restaurants. During a visit on a typical working day, we observed interactions between waitstaff and the delivery robot over several hours. These observations provided valuable contextual insight into the robot’s practical functioning and the ways staff engaged with it. Although not treated as a primary data source, the observational material enriched our interpretation of the interview data by offering a clearer understanding of the work routines, spatial dynamics, and interaction patterns described by participants. During this visit, we also interviewed two employees who shared valuable insights into the robot’s role in their everyday work and their experiences collaborating with it. Each interview in the second part of the study lasted between 30 and 40 min. Table 1 provides an overview of the interviews conducted, i.e., twenty-two interviews with thirty-four interviewees across individual, paired, and group formats.

**TABLE 1:** Interview summary.

Study part	Interviewees	Number of participants	Interview format	Number of interviews
First study part	Four guests, one restaurant manager, five project managers	10	Single interviews	10
	Eight guests, two restaurant managers, two project managers	12	Paired (groups of two)	6
	Eight guests	8	Two group interviews (four participants each)	2
Second study part	One restaurant owner, one robot supplier, two waitstaff employees	4	Single interviews	4
Total		34		22

### 3.3 Data analysis

To systematically categorize and interpret the data, we applied coding. We began with a deductive approach, using Baškarada et al.’s (2013) four stages of technology deployment, i.e., project initiation, planning, execution, and evaluation, as initial categories. This was followed by an inductive process, allowing themes to emerge from the data. Table 2 provides a structured view of the themes and subthemes that emerged associated with the four project stages.

**TABLE 2:** Thematic structure of findings.

Project Stage	Themes	Subthemes
1. Project initiation	Investment aspects	Health and safety; staffing shortages; workplace inclusion; cost efficiency; customer service; expertise
	Resistance aspects	Unemployment concerns; communication; trial period
2. Project planning	—	Facility requirements; integration during construction; future orientation; learning from others
3. Project execution	—	Understanding operations; user involvement; new mindsets; human–machine interaction; adjustment processes; alignment between robot and workflow
4. Project evaluation	Lessons learned	Physical relief; novelty effects; efficiency outcomes; retraining

### 3.4 Method assessment

Research quality is typically assessed through validity and reliability, with external validity concerning the generalizability of findings (Yin, 2015). Because this study examines a specific type of delivery robot in Norwegian restaurants, caution is needed when extending the results to other contexts or robot types. A key reliability concern relates to participant sampling. Although guests, managers, suppliers, and waitstaff were included, professional interviewees were weighted toward managerial roles, which may have encouraged more positive assessments of the deployment. Social desirability bias, defined as a tendency to answer in a socially acceptable direction (Lewis-Beck et al., 2003), may also have influenced employee responses when discussing new technology. A second concern involves interview accounts of reduced workload and physical strain. Although interviewees emphasized less walking back and forth between tables and the kitchen while carrying heavy plates, these descriptions may oversimplify the overall workload implications, as the study did not assess the effort required for tasks such as loading the service robot.

To strengthen reliability, we combined interviews with direct observations and included participants in varied roles. Our observational study enabled us to get estimates of the weight of plates carried and the walking distance saved, although we were unable to examine loading and unloading in different operational scenarios. The data may therefore still reflect role-specific perspectives and varying comfort levels in expressing critical views. Although the study identifies important aspects across initiation, planning, execution, and evaluation, it does not claim to provide a complete account of service robot deployment. The findings should therefore be viewed as contextual insights that contribute to, but do not fully define, understanding of service robot deployment in hospitality settings.

## 4 Results

Table 2 in Section 3.3 lists the key themes that emerged from our data, aligned with the four project stages that guided our analysis. This section presents the study’s findings. Section 4.1 synthesizes the main aspects identified in response to RQ1 into a summary table (Table 3) and a flow chart, while Section 4.2 presents the findings related to RQ2 as a numbered list supported by a flow chart (Figure 3), thereby improving thematic coherence and readability. For RQ1: *What are the key aspects of service robot deployment, from project initiation through evaluation?* Section 4.3 provides detailed findings for each project stage: project initiation (4.3.1), project planning (4.3.2), project execution (4.3.3), and project evaluation (4.3.4). For RQ2: *What approach is used to ensure that robot functionality aligns with restaurant workflows and spatial configurations?* A detailed answer is presented in Section 4.3.3.

**TABLE 3:** Key aspects identified in response to RQ1.

Project initiation
- Investment aspects:
○ Health and safety: Protecting workers’ wellbeing is a key motivation, as staff are viewed as the restaurant’s most valuable asset.
○ Staffing challenges: Labor shortages drive service robot adoption
○ Workplace inclusion: Robots can support workplace inclusion by reducing physical demands
○ Cost efficiency: Robots provide cost efficiency through their uninterrupted and reliable operation, and they may help lower sick-leave rates by undertaking physically strenuous tasks
○ Customer service: Robots may allow staff to focus more on guest interaction
○ Expertise: Domain-specific expertise of human staff remains irreplaceable
- Resistance aspects:
○ Unemployment concerns: Robots are often met with fears of contributing to broader societal unemployment.
○ Communication: Clear communication is essential to foster understanding of the robot’s role and reduce skepticism
○ Trial period: Allowing time to test the robot’s value can help mitigate resistance
Project planning
○ Facility requirements: Effective robot use depends on a work area layout that supports navigation
○ Integration during construction: Considering robot integration during the initial restaurant construction phase can facilitate smoother implementation
○ Future focus: Planning should balance technological foresight with practical execution
○ Learning from others: Studying successful implementations in other restaurants can offer valuable insights
Project execution
○ Understanding operations: Robot behavior must align with workflows, complementing staff, and used only where they outperform humans
○ User involvement: Staff should be involved in decisions about robot use and movement
○ New mindsets: Successful integration requires a shift in employee mindset.
○ Human-machine interaction: Managing the dynamic between robots and staff is crucial
○ Adjustments: Testing and staff feedback are essential for tailoring robot behavior to the restaurant’s concept
○ Alignment process: For details on steps followed to align robot functionality with workflows and spatial configurations, see Section 4.3.3
Project evaluation
- Lessons learned:
○ Physical relief: Robots reduce physical strain, potentially lowering sick leave and early retirement rates.
○ Efficiency: Robots are especially helpful in unplanned high-volume situations, such as large, unbooked table settings
○ Novelty: Food service robots introduce a sense of novelty to the dining experience, especially during children’s birthday celebrations
○ Retraining: “Action point”: Staff must be retrained to adapt to new workflows involving robots

### 4.1 Summary: key aspects of deployment from project initiation to evaluation (RQ1)

**FIGURE 2:**
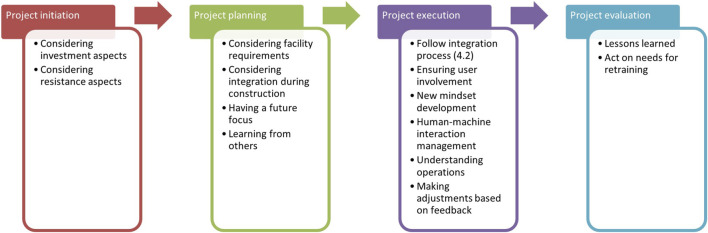
Deployment aspects, from project initiation to evaluation.

This summary distills the key aspects identified in response to RQ1 into a table, Table 3, and a flow chart, Figure 2.

### 4.2 Summary: integration into restaurant operations (RQ2)

**FIGURE 3:**
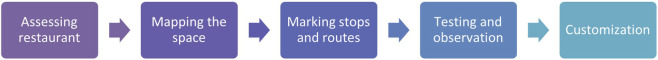
Integration process.

According to the robot supplier, the integration process (being part of project execution) is designed to align robot functionality with restaurant workflows and spatial configurations through a series of practical steps. This summary distills the findings identified in response to RQ2 into a numbered list and a flow chart, Figure 3.

1. **Assessing Restaurant Facilities:** A thorough evaluation of the restaurant layout is conducted to identify obstacles and areas requiring adaptation for smooth robot navigation.
2. **Mapping the Space:** The robot is guided through the restaurant to generate a spatial map. Collected data is processed digitally to create a navigable model of the environment.
3. **Marking Specific Stops and Routes:** Specific locations for robot stops are marked, and operational routes are defined within the digital map.
4. **Testing and Observation:** The robot is deployed on-site for a trial period, during which its performance is observed, and feedback is gathered from staff. Adjustments are made if spatial constraints hinder movement.
5. **Customization:** Final refinements are applied to tailor the robot’s behavior to the restaurant’s operational needs, including personalized features such as welcome messages and birthday songs.

### 4.3 Detailed findings

In the following section, we present detailed findings related to RQ1, organized according to the four project stages: project initiation (Section 4.3.1), project planning (Section 4.3.2), project execution (Section 4.3.3), and project evaluation (Section 4.3.4). With respect to RQ2, the approach used to ensure alignment between robot functionality and restaurant workflows and spatial configurations is detailed in Section 4.3.3.

#### 4.3.1 Project initiation

Several aspects were identified as potentially influencing the decision to utilize service robots. Restaurant managers highlighted *staffing shortages* as a key motivation for initiating projects involving service robots. However, they emphasized that their intention was not to replace their staff. Instead, they opted to introduce delivery robots as time-saving tools for their employees. Managers suggested integrating heating cabinets into the robots to assist waiters in serving warm food to multiple guests simultaneously. In the interview with the restaurant owner, the central focus was on *health and safety* considerations for employees. The owner emphasized that employees who interact directly with guests are of utmost value to the restaurant. Unfortunately, working in restaurants is physically demanding, making it crucial to investigate ways to reduce the wear and tear on employees. The owner reflected, “*When the possibility of implementing robots in the workplace arose, it was therefore not difficult to give it a try*.” Additionally, managers emphasized the importance of health and safety. Around half of the interviewees acknowledged the positive role service robots can play in reducing the risk of repetitive strain injuries in the restaurant environment. They emphasized that robots could assist with physically demanding carrying tasks, thereby helping to prevent injuries to employees’ shoulders, backs, and feet. By delegating such repetitive tasks to robots, employees would also be able to devote more time to direct customer service.

Importantly, the interviewees did not support replacing human workers with robots. Instead, they suggested that employees should be relieved of monotonous tasks, such as cleaning and carrying, so they can focus on more value-creating, customer-facing responsibilities. Several participants noted that having a robotic “colleague” could help reduce workload and contribute to a healthier working environment. One manager, who actively worked front-of-house in his restaurant, illustrated the physical toll of the job: “*After working 12–14 h, I am too tired in my arms to even lift my little three-year-old girl*.” As regards workplace inclusion, one waiter noted, “*It goes without saying that here too there are savings in terms of waiters not having to carry as heavy as without robot assistance*.” The waiter added, “*One additional benefit of the robots’ capacity to lift heavy objects is that in the future, hiring procedures will not need to focus as much on choosing candidates with strong physical attributes, which again is an aspect of workplace inclusion at restaurants*.”

Pertaining to *cost efficiency*, interviewees reflected on the expectation that service robots could help reduce physical strain on staff. Managers and several guests emphasized an important trade-off: although robots require an upfront investment, many considered this cost to be outweighed by long-term benefits, including reduced physical load on employees, improved workplace wellbeing, and the possibility of lower sick-leave rates. From this perspective, robotic assistance was viewed as a strategic investment that supports employee health and stable operations. At the same time, approximately one-fourth of the guests pointed to a different economic rationale. They highlighted the productivity advantages of robots and noted that robots are always available, can work continuously, and do not complain. From this viewpoint, robots were seen as potentially highly cost effective because of their consistent availability and reliable performance. Managers recognized the added benefit of having robots carry food to tables, allowing waiters to focus on increasing sales by promoting more wines and beverages, thereby enhancing overall *customer service*. Additionally, when arranging large dinner parties or banquets, managers saw immense potential in robots transporting large quantities of food from the kitchen to waiters, who would then be ready to serve guests at the tables.

When interviewing on potential service robot use cases and contributions, interviewees consistently highlighted that robots cannot replace *expertise* of human workers in their specific domains. A common sentiment was that robots should only be implemented if they can execute tasks with greater precision than humans. However, opinions varied regarding the practical and valuable contributions robots can make. One interviewee acknowledged that while employment opportunities will persist for individuals with varying levels of education, the dynamics of work will evolve, and the skills valued in today’s jobs may not be as important in future roles. When discussing *unemployment fear* in terms of robots potentially replacing staff, nearly half of the interviewed guests expressed concerns. Specifically, they worried about the impact on students, immigrants, and individuals with limited education, with a main fear that widespread robot adoption will lead to unemployment in these vulnerable groups. Some informants reflected that whilst technological developments have historically resulted in the displacement of human jobs, they have also generated new work prospects.

Given that humans are often skeptical of recent technology, the supplier noted that effective *communication* is essential when deploying service robots in the workplace to address this skepticism. In this respect, the supplier emphasized the importance of presenting the robot as a “*carrying aid*”, “*an automated trolley*”, and “*a health, environment, and safety initiative*”. This is particularly relevant in a sector facing personnel shortages and high sick leave rates. By doing so, it helps to prevent opposition from people who might fear robots taking over jobs and conquering the world, as portrayed in books and movies. The supplier explained that customers need both time and hands-on experience to fully understand the robot’s value. “*Rather than brief demonstrations, we first show potential clients the robot operating in a real restaurant. We then offer them a trial period in their own workplace. This allows them to see how the robot reduces heavy carrying tasks and frees waitstaff to spend more time with guests.*” From previous sales, the supplier had observed that when customers are not given enough time to evaluate the robot, misconceptions can arise, such as concerns about robots replacing human labor. “*Offering a trial period helps prevent these misunderstandings and allows customers to see the robot for what it is: a health, environment, and safety measure that reduces strain injuries and can even help lower sick leave*,” the supplier said, adding that “*letting customers try before they buy has proven to be a successful approach*.”

#### 4.3.2 Project planning

Pertaining to *facility requirements*, the supplier emphasized that many dining establishments lack the necessary facilities to effectively accommodate and operate the service robot. “*If the robot is to be used, there needs to be a waiter-operated area that is at least sufficiently large*”, he said. According to the restaurant owner, the restaurant we visited was an obvious first choice for easy implementation, as it is spacious and requires no architectural modifications for the robots to navigate. Restaurant managers noted that robots need regular charging and sufficient space, without floor levels and doorsteps, to move freely. Regarding the aspect of *integration during construction*, the owner emphasized that compared to the restaurant we visited, “*In other restaurants, stairs, artificial plateau designs, high doorsteps, and other architectural conditions may require rebuilding, depending on economic and structural considerations*.” Managers noted that if they had considered robot integration during construction, they would have designed the restaurant differently to ensure smooth robot implementation, thereby avoiding subsequent remodeling expenses. Whilst managers acknowledged that effective planning requires having a *future focus*, they also emphasized the importance of considering potential negative effects of implementing new and costly technological solutions that can quickly become outdated. Balancing foresight with practical project execution is a delicate decision-making aspect. As a result, some may choose to wait for more mature technological advancements rather than rushing into untested innovations. As regards *learning from others*, interviewees acknowledged the importance of examining prosperous companies that keep up with technological advancements and can identify and consider technological solutions, such as service robots, early in a project, to learn from their experiences.

#### 4.3.3 Project execution

Regarding *understanding operations*, the supplier emphasized that managing the interaction between robots and humans is essential, and success lies in understanding the dynamic interaction between the robot and the waiter, i.e., understanding operations. “*The goal is to tailor the robot’s procedures to fit the restaurant’s concept*”, he said. He emphasized that in this respect it is important to keep in mind that the focus is on getting the robot to support the workers, not replacing them, and that robots should only be used if they perform tasks with greater precision than humans. The supplier emphasized *user involvement* as a central aspect. Decisions about robot movement should be made by those working with it. “*In this respect it is central to create a sufficient understanding of what the robot can do*”, he said. The supplier also emphasized that it is vital for employees to adopt a *new mindset* when working alongside robots. In this regard, it may take some time for waiters to adapt to a work process where robots are optimally utilized.

During discussions with the supplier about project execution, how food service robots are *aligned with* restaurant operations became a focus. According to the supplier, addressing **RQ2**, the technological deployment of service robots in restaurants follows a relatively straightforward *integration process*: It begins with an on-site assessment of the restaurant’s physical environment to identify potential obstacles and areas that may require adaptation for smooth robot navigation. Once this assessment is complete, the supplier manually guides the robot through the restaurant to generate a map of the space. A computer is used to process this generated map. Specific stopping points and routes for the robot to follow are marked on the map. As part of testing, the supplier noted that tightly spaced tables can hinder robot navigation. Over a few days of live use, staff feedback is gathered to *adjust* robot behavior, such as movement patterns, welcome messages, and birthday songs, to suit the restaurant’s needs. With regards to putting the robot into action, the supplier stressed the need for a deep understanding of restaurant operations to tailor its behavior to the dining experience. Understanding and managing the intricacies of *human-machine interaction*, which align with the restaurant’s concept, is crucial. “*At the restaurant studied, waiters serve the food after the robot brings it to its designated location, which differs from setups where robots deliver dishes directly to guests for self-service*”, the supplier emphasized. Regarding usability, the supplier highlighted that the robot’s interface is intentionally simple and can be operated with a single button. This design allows staff to adapt quickly and makes the robot a practical support tool for tasks such as transporting food to tables.

#### 4.3.4 Project evaluation

A theme among managers was the need for *retraining* of waiters. Some initial skepticism toward technology had been observed. Managers emphasized the need for a shift in mindset wherein waiters recognize that tasks such as carrying food and dishes can be effectively managed by robots. One manager explained that while kitchen and bar routines are designed to minimize staff movement, waiters traditionally operate under the principle of never leaving a table empty-handed. To fully integrate robots into the workflow, retraining is essential. This involves empowering waiters to adopt new ways of thinking and working. The supplier echoed this point, stressing that adapting to a new service model is key to leveraging the robot’s capabilities.

The *physical relief* benefits of robotic assistance were demonstrated during field observations. In collaboration with a waiter, a practical example was calculated: the robot can carry eight plates per trip, each weighing approximately 2.2 kg. To serve a large table of 20 guests, the robot completes two trips (16 plates), while the waiter carries the remaining four plates in two trips. Without the robot, the waiter would have needed to make ten round trips to serve all 20 plates. Thanks to the robot, this is reduced to just two trips, resulting in a total of 320 m of walking saved. The robot also carries a total of 35.2 kg, significantly reducing the physical load on the waiter. One staff member reflected that this reduction in physical strain could help prevent long-term wear and tear on the body, potentially lowering the risk of sick leave and early retirement. The robot was also noted to be particularly helpful during birthday parties, where it often carries multiple pizzas, each plate weighing around 1.2 kg, further easing the workload on staff.

Regarding *efficiency*, waiters reported that the robot is especially useful during peak times or when serving large groups over long distances. One waiter noted that in situations where large tables are not booked in advance, the robot becomes a valuable asset, as there may not be time to bring in additional staff. However, in areas close to the kitchen, it can sometimes be quicker for the waiter to serve guests directly rather than using the robot. This highlights the importance of context in determining the robot’s efficiency.

Guests generally described their encounters with the robot as entertaining, modern, and unique, adding a sense of *novelty* to the dining experience. However, they also emphasized the continued importance of human interaction. Conversations, eye contact, and personalized recommendations from waiters were seen as essential elements of hospitality that robots cannot yet replicate. Regarding *robot popularity*, waiters reported that the robots have become well liked among guests, particularly during children’s birthday celebrations. They noted that the robot’s ability to sing birthday songs and assist with food delivery in an entertaining and playful manner has made it a highlight of the dining experience for younger guests.

## 5 Discussion

In response to calls by Madhan et al. (2023) and Xu et al. (2023) for more real-world case studies, our findings provide grounded insights from pioneering Norwegian restaurants, covering the deployment process, from project initiation to post-implementation. In relation to **RQ1**, a broad spectrum of deployment-related aspects has been identified and presented throughout Section 4.3 with a concise summary provided in Section 4.1 for ease of reference. For **RQ2**, the in-depth interview with the robot supplier offered detailed insights into the approach used to align robot functionality with restaurant workflows and spatial configurations, as summarized in Section 4.2 and detailed in Section 4.3.3.

At the **project initiation** stage, understanding both the motivations for adopting robots and the sources of hesitation is critical (Ho and Chuah, 2025), as these factors significantly influence decisions about whether to proceed with deployment (Mitish et al., 2021). Our findings reveal a spectrum of attitudes toward service robotics, shaped by both investment considerations and potential resistance. Notably, managers generally expressed positive views, for instance emphasizing robots as a viable solution to staffing shortages, an outlook that contrasts with earlier studies by Seyitoğlu et al. (2021), which reported predominantly negative managerial attitudes. While waiters who interacted directly with the robots described several benefits, particularly reduced walking and carrying tasks, concerns were raised by guests, especially regarding potential job displacement for individuals with limited or no formal education. Similar concerns were echoed among hotel staff in the study Vatan and Dogan (2021).

An interesting insight emerging from our empirical material concerns how the robot supplier strategically framed the technology when presenting it to potential customers. The supplier consistently described the robot as a carrying aid, an automated trolley, and a health, environment, and safety initiative. This framing served to emphasize the robot’s supportive and practical role rather than suggesting that it might replace human labor. The supplier’s use of such mechanical metaphors brings to mind Gareth Morgan’s widely recognized classic *Images of Organization* (Morgan, 1998), even though this literature was not part of our formal review. Morgan’s work is well known for demonstrating how metaphors shape organizational sensemaking by directing attention toward certain interpretations while downplaying others. In this case, the supplier’s framing highlights the importance of reflecting on how service robots are presented to potential users, whether as functional tools or as human-like collaborators, since such presentations may influence perceptions and acceptance during the adoption process.

The extent to which the robots appeared to alleviate the physical burden on waiters was notable; however, the study was limited in its capacity to comprehensively assess workload implications over the course of an entire work shift, including cumulative physical strain and the total physical effort associated with loading and unloading the robot. Within these constraints, interviewees consistently described reduced walking distances and less frequent carrying of heavy plates as substantial benefits. In addition, the findings point to a dimension that has received limited attention in existing literature: food service robots may contribute to workplace inclusion by lowering the emphasis on strong physical attributes in service staff. This is particularly salient in the Norwegian context, where the Inclusive Working Life (IA) Agreement seeks to broaden participation by enabling individuals who can and wish to contribute to remain in or enter the workforce. It also resonates with the recruitment and retention challenges in the restaurant sector highlighted by Olsson (2024).

Investment-related considerations were also prominent in the data. In this respect, although the findings of this study show that service robots provide several operational advantages, including continuous operation, support in reducing employees’ workload, and overall ease of use, these benefits must be weighed against the economic conditions required for such an investment. From an economic perspective, McLaney et al. (2007) and Berk and DeMarzo (2017) emphasize that the adoption of new technology can only be justified when the investment is expected to improve a company’s financial performance and create value for its owners. Ivanov and Webster (2019b) similarly argue that a lack of clear economic gains reduces the incentive to adopt new technologies such as service robots. In this context, it is relevant that the initial cost of service robots may be perceived as high compared with more traditional and less expensive equipment, such as an industrial dishwasher. This can complicate the economic assessment, especially for businesses operating with tight margins. At the same time, recent research shows that the motivation to adopt service robots is often linked to efficiency improvements and long-term cost reductions (Belanche et al., 2021; Skubis et al., 2024). Furthermore, the market has evolved in recent years, offering a wider variety of robot models and price levels. This increased variety enables companies to align potential investments more closely with their financial capacity and operational requirements. Consequently, the economic barrier to adoption may be reduced, making technology more accessible and, in some cases, more financially attractive for a wider set of businesses. At the same time, respondents frequently noted uncertainties related to the rapid pace of technological development and the risk of investing in a solution that may quickly become outdated. These concerns highlight that the financial reasoning behind robot adoption is multifaceted and shaped not only by upfront costs and anticipated benefits but also by perceived technological and investment risks.

Regarding **project planning**, interviewees emphasized the importance of learning from previous implementations and considering robot integration early in the design of new restaurants. Planning ahead ensures that the physical environment supports robotic functionality, avoiding costly retrofits. This approach reflects systems engineering principles, which advocate for a structured and holistic design of complex enterprise systems (Kossiakoff et al., 2020).

During **project execution**, the supplier stressed the importance of understanding restaurant operations and aligning robot behavior with existing workflows. This reflects findings on enterprise-digital technology alignment (Kaidalova et al., 2014). A surprising finding in this context was the simplicity of the robot mapping process: the robot was manually guided through the space to generate a digital map, which was then processed by computer software. This contrasts with traditional business process modeling, often described as time-consuming and complex (Alotaibi, 2016), with BPMN as the standard notation (Compagnucci et al., 2024).

Managers also emphasized the need for employees to adopt new mindsets, as job roles are likely to evolve. This supports Neeley and Leonardi’s (2022) argument that employees must develop attitudes and behaviors that allow them to see how data, algorithms, and AI can create new opportunities. The importance of user involvement in robot deployment aligns with longstanding findings that user participation improves system success (Bano and Zowghi, 2015). Moreover, the view that robots should not replace human workers is consistent with research showing that roles requiring high levels of interpersonal interaction cannot be automated (Mingotto et al., 2021).

Regarding **project evaluation**, various lessons learned could be noted. The deployment of the service robots was found to reduce physical strain on waiters, particularly during peak hours and when serving large tables. An additional benefit was the novelty the robots introduced to the dining experience, especially during children’s birthday celebrations. In relation to Ho and Chuah’s (2025) finding that openness to new experiences influences willingness to work with robots, our study did not observe such a connection. However, the need for staff retraining to adapt to new technologies was evident and aligns with the conclusions of both Takagi et al. (2025) and Ho and Chuah (2025), who emphasize training as essential to realizing the full benefits of technology investments.

## 6 Conclusion and further work

### 6.1 Theoretical implications

This study advances theoretical understanding of service robot deployment by addressing key gaps in existing literature. First, it adopts a holistic perspective by incorporating insights from both technology providers and frontline employees, offering a more comprehensive view of deployment dynamics. Second, the research spans the full project lifecycle, from initiation through planning, execution, and evaluation, thereby contributing to a more nuanced understanding of the phased nature of technological change in service environments. This broader scope supports the refinement of theoretical models that account for the complexity and interdependencies inherent in technology integration projects. Third, the study responds to the geographic concentration of prior research, which has predominantly focused on cases from the People’s Republic of China and the United States. By examining early adoption in Norwegian restaurants, a technologically progressive yet underexplored context, the study introduces a novel empirical setting that extends the geographical and cultural scope of existing research. This contribution opens avenues for comparative studies exploring how contextual factors, including national regulations, labor market conditions, and cultural attitudes, influence the adoption and integration of service robots.

### 6.2 Practical implications

The findings of this study offer practical guidance for professionals considering the adoption of service robots in restaurant environments. By examining the full deployment process, from project initiation through to evaluation, the study provides a detailed view of the conditions, challenges, and potential outcomes involved in integrating delivery robots into daily operations. It also sheds light on broader implications, such as addressing labor shortages and reducing the physical demands placed on waitstaff. Importantly, the research emphasizes the value of incorporating service robots during the early design and construction phases of new restaurants. Doing so can facilitate smoother implementation and help avoid costly modifications later. Overall, the study underscores the importance of a structured, end-to-end approach to technology deployment, one that integrates spatial planning, workforce readiness, and customer experience right from the start to ensure everything works together smoothly.

### 6.3 Further research

This exploratory–explanatory study offers contextual insights into how service robots are introduced and integrated into restaurant operations. Building on these findings, several directions for further work naturally follow from the empirical patterns observed. Future studies could examine the physical work associated with robot-supported service in greater detail, including tasks such as loading the robot and maneuvering it in different operational conditions. Our observations provided useful indications of distances walked and weights carried, and more systematic measurement could help clarify how physical strain is distributed across the full work process. Expanding the number of interviews with waitstaff who work directly with service robots would also be valuable. Their experiences, including moments of adaptation or challenge, can provide important perspectives that complement managerial and customer viewpoints. It may additionally be useful to explore how different service configurations influence both staff workload and customer experience. For example, whether waiters serve food from the robot or guests help themselves could shape perceptions of usefulness, workflow efficiency, and interaction patterns. Also, the tension between the robot’s appealing anthropomorphic/bionic features and the need to clarify its practical role as a carrying assistant may offer a meaningful direction for further research. Comparative studies across national contexts could also enrich understanding. Much existing research on restaurant service robots stems from other regions, and contrasting such studies with insights from the Norwegian setting examined here may shed light on how cultural and institutional factors influence expectations and acceptance. Additionally, further attention to front-end planning appears promising. Investigating how restaurants incorporate potential robot use into the early design of facilities could yield insights into how to streamline implementation and reduce subsequent adaptation costs, particularly as service robots become more common in hospitality environments. Finally, from a broader perspective, introducing service robots can drive both process, product, and business-model transformation. While robots may streamline employee tasks and add novelty to the service experience, they can also prompt business-model shifts when assuming full-service roles. Advances in robotics therefore raise questions about their responsible integration into service ecosystems, particularly regarding the future distribution of work and ongoing debates about whether robots should replace or complement human labor. These concerns, echoed by several interviewees, highlight the need for continued research and informed debate on how service robots should be deployed in hospitality operations and what this means for workforce roles and the long-term sustainability of service work.

## Data Availability

The original contributions presented in the study are included in the article/supplementary material, further inquiries can be directed to the corresponding author.
